# CELEBRATING THE 100TH DAY OF SCHOOL: PILOTING AN AGEISM-REDUCING TOOLKIT FOR PREK TO SECOND GRADERS

**DOI:** 10.1093/geroni/igae098.3825

**Published:** 2024-12-31

**Authors:** Tina Newsham, Cynthia Hancock, Daniel Alston, Lisa Borrero, Katherina Nikzad-Terhune, Sarah Tesar, Elizabeth Fugate-Whitlock

**Affiliations:** University of North Carolina Wilmington, Wilmington, North Carolina, United States; University of North Carolina Charlotte, Charlotte, North Carolina, United States; University of North Carolina Charlotte, Charlotte, North Carolina, United States; University of Indianapolis, Indianapolis, Indiana, United States; Northern Kentucky University, Lexington, Kentucky, United States; University of North Carolina Charlotte, Charlotte, North Carolina, United States; University of North Carolina Wilmington, Wilmington, North Carolina, United States

## Abstract

Upon learning that some celebrations of the 100th day of school involve young children dressing up “like a 100-year-old,” our team questioned the ageist ideas this activity could reinforce. Children adopt beliefs about various social groups at an early age. They are inundated with ageist messages through books, TV, and other sources and internalize those ideas without questioning them. Those who hold negative views of aging live, on average, 7.5 fewer years than those who hold positive views. To prevent negative outcomes, children must be taught accurate and positive information about aging and older adulthood from an early age. The purpose of our research project is to provide teachers with evidence-based educational materials related to aging (centenarians in particular). We created a toolkit about aging and ageism to give PreK and elementary teachers options for celebrating the 100th day of school in a way that reinforces academic content while supporting age-inclusivity. The toolkit includes lesson plans celebrating centenarians. We used a mixed-method approach to pilot test the toolkit with 40 teachers and obtained a complete data set from 25 of them. Quantitative data were collected pre- and post-pilot using the 12-item Expectations Regarding Aging Survey. Qualitative data were collected through online pre-and post-pilot survey questions and post-pilot semi-structured interviews. We provide details on the toolkit and findings from the pilot study, which revealed a statistically significant improvement in expectations about aging (p< 0.05) and strong qualitative support for the value of the toolkit.

